# Sustained Inflation at Birth Did Not Alter Lung Injury from Mechanical Ventilation in Surfactant-Treated Fetal Lambs

**DOI:** 10.1371/journal.pone.0113473

**Published:** 2014-11-24

**Authors:** Noah H. Hillman, Matthew W. Kemp, Yuichiro Miura, Suhas G. Kallapur, Alan H. Jobe

**Affiliations:** 1 Division of Neonatology, Cardinal Glennon Children's Medical Center, Saint Louis University, Saint Louis, Missouri, United States of America; 2 Division of Pulmonary Biology, Cincinnati Children's Hospital Medical Center, University of Cincinnati, Cincinnati, Ohio, United States of America; 3 School of Women's and Infants' Health, University of Western Australia, Perth, Western Australia, Australia; University Children's Hospital Basel, Switzerland

## Abstract

**Background:**

Sustained inflations (SI) are used with the initiation of ventilation at birth to rapidly recruit functional residual capacity and may decrease lung injury and the need for mechanical ventilation in preterm infants. However, a 20 second SI in surfactant-deficient preterm lambs caused an acute phase injury response without decreasing lung injury from subsequent mechanical ventilation.

**Hypothesis:**

A 20 second SI at birth will decrease lung injury from mechanical ventilation in surfactant-treated preterm fetal lambs.

**Methods:**

The head and chest of fetal sheep at 126±1 day GA were exteriorized, with tracheostomy and removal of fetal lung fluid prior to treatment with surfactant (300 mg in 15 ml saline). Fetal lambs were randomized to one of four 15 minute interventions: 1) PEEP 8 cmH_2_O; 2) 20 sec SI at 40 cmH_2_O, then PEEP 8 cmH_2_O; 3) mechanical ventilation with 7 ml/kg tidal volume; or 4) 20 sec SI then mechanical ventilation at 7 ml/kg. Fetal lambs remained on placental support for the intervention and for 30 min after the intervention.

**Results:**

SI recruited a mean volume of 6.8±0.8 mL/kg. SI did not alter respiratory physiology during mechanical ventilation. Heat shock protein (HSP) 70, HSP60, and total protein in lung fluid similarly increased in both ventilation groups. Modest pro-inflammatory cytokine and acute phase responses, with or without SI, were similar with ventilation. SI alone did not increase markers of injury.

**Conclusion:**

In surfactant treated fetal lambs, a 20 sec SI did not alter ventilation physiology or markers of lung injury from mechanical ventilation.

## Introduction

In order to initiate gas exchange, the newborn infant must quickly clear fetal lung fluid from the airways and establish a functional residual capacity (FRC). Using large, negative pressure breaths, the normal newborn pulls the lung fluid from the airways into the distal airspaces and parenchyma, and clears this fluid over subsequent minutes to hours [1], [2]. Since many very low birth weight (VLBW) preterm infants are surfactant deficient and lack the respiratory muscles to overcome the high surface tension of fluid in the lungs, FRC is difficult to accumulate and maintain. To overcome the long time constants created by the air-fluid interfaces in small airways, physicians have begun to use initial prolonged inspiratory times to recruit FRC and to more uniformly aerate the fluid-filled preterm lung, commonly referred to as a sustained inflation (SI) [1], [3]–[5]. A SI at birth decreased the need for mechanical ventilation at 72 hours and may lead to a decrease in bronchopulmonary dysplasia (BPD) [4], [6]. Although the European guidelines for newborn resuscitation suggest five, 2 to 3 second SIs may be helpful in VLBW infants, other studies have suggested a longer SI time is necessary to recruit FRC [4], [5], [7]. A multi-national randomized, controlled clinical trial is underway using 15 second SI at birth in VLBW (NCT02139800). The rapid movement of lung fluid in the airways during the initiation of ventilation also contributes to sheer force injury to the epithelium [8]. Therefore, it is important to determine if recruitment of FRC with SI is safe.

Animal studies have provided important information about lung recruitment, physiologic responses, and potential injury from a SI at birth. In fetal sheep and preterm rabbits, of the volume recruited by a SI over 50% was recruited by 5 seconds, and 90% by 15 seconds [9], [10]. Although pressure-limited or volume-limited SI maneuvers recruited similar FRC, there were no differences in markers of lung injury between the two methods, and pressure-limited SI is more practical in the delivery room [11]. A SI at birth augmented the cardiorespiratory transition in preterm lambs and improved the heart rate response to resuscitation of asphyxiated near-term lambs [3], [12]. However, a rapid rise in HR after SI has yet to be found in preterm infants [7]. Although a SI recruited a variable FRC in surfactant deficient lambs, markers of lung injury with subsequent ventilation did not change with FRC [10]. Of some concern, SI alone modestly increased markers of lung injury [10]. Small amounts of endogenous surfactant will decrease injury from mechanical ventilation [13], [14]. Surfactant treatment also decreases markers of lung injury from mechanical ventilation at birth and improves the spatial distribution of air from a SI [15], [16]. Therefore, we have tested the hypothesis that a 20 second SI would recruit FRC and decrease lung injury caused by ventilation of the surfactant treated preterm sheep lung.

## Methods

The Animal Ethics Committees of the University of Western Australia, Cincinnati Children's Hospital Medical Center, and Saint Louis University approved the studies.

### Fetal exposure for ventilation

Date mated Merino Ewes at 126±1 days gestational age (term is approximately 150 days GA) were premedicated with xylazine (0.5 mg/kg) IM, ketamine (5 mg/kg) and midazolam (0.25 mg/kg) IV. Ewes were intubated and anaesthesia maintained with isofluorane (0.5–2% in 100% O_2_), which crosses the placenta and anesthetizes the fetus [10]. The fetal head and chest were exteriorized through a midline hysterotomy, while maintenance of placental blood flow [17]. The fetus received a tracheostomy to secure a 4 F endotracheal tube. Free-flowing fetal lung fluid (FLF) was gently removed with an 8 F catheter (average 30 mL) and an aliquot was snap frozen. Surfactant (300 mg (Curosurf Chiesi S.p.A., Italy) diluted in 15 mL normal saline was instilled into lung via ET tube, gently aspirated for mixing, and returned to the lung prior to ventilation to deliver a dose of 100 mg/kg surfactant based on estimated fetal weight of 3 kg.

### Physiologic measurements

The endotracheal tube was attached to a Fabian ventilator (Acutronic, Hirzel, Switzerland) and a Neopuff (Fisher & Paykel, Auckland New Zealand) via a three-way slip valve (Hans Rudolph, Kansas City, MO) to avoid loss of FRC when changing from SI to mechanical ventilation (MV). In line with the endotracheal tube, we placed the flow sensor for the Fabian ventilator and Hans Rudolph 1.3 mL pneumotach (RSS HR100 system, Hans Rudolph, Kansas City, MO). The Han Rudolph system was attached to computer to continuously record the volume recruitment over time. HR volumes previously correlated with total volumes recorded with respiratory impedence bands [10].

### Fetal Interventions

The fetal lambs were randomly assigned to one of four groups for the 15 minute intervention: 1) a PEEP of 8 cmH_2_O (PEEP); 2) a 20 seconds SI at 40 cmH_2_O with a T-piece resuscitator (Neopuff) using a flow-rate of 8 L/min, followed by PEEP of 8 cmH_2_O (SI); 3) mechanical ventilation with a tidal volume (V_T_) of 7 ml/kg from birth (V_T_); or 4) 20 seconds SI at 40 cmH_2_O then mechanical ventilation (SI+V_T_). The fetal lambs randomized to V_T_, with or without SI, were ventilated at 40 breaths/min with an inspiratory time of 0.7 seconds. The Fabian ventilator was set for IPPV-volume guarantee at 7 mL/kg based on estimated fetal weight, with a maximal PIP of 40 cmH_2_O. Peak pressures and tidal volumes were recorded to determine lung compliance during the 15 min ventilation period. All lambs received heated, humidified 95% N_2_ and 5% CO_2_ gas mixture to avoid oxygen exposure and changes in fetal PCO_2_. After the intervention, the endotracheal tube was occluded and the fetus was maintained on placental circulation for additional 30 minutes. At 45 minutes from the beginning of the recruitment procedure, the fetus was euthanized with pentobarbital (100 mg/kg), and fetal lung fluid was collected and snap frozen (FLF end). Cord blood gasses were measured using a Siemens Rapidlab 1265 (Siemens, Bayswater, Australia). The 45 minute experimental period was chosen to optimize the detection of mRNA for acute phase inflammatory responses [18]. Based on power calculations from previous experiments [10], [15], smaller groups were used for the PEEP control (n = 4) and SI alone (n = 6) to conserve animals.

### Lung Processing and BAL Analysis

At autopsy, a deflation pressure-volume curve was measured from an inflation pressure of 40 cmH_2_O pressure [19]. Bronchioalveolar lavage fluid (BALF) of the left lung was collected by repetitive saline lavage. FLF and BALF were used for measurement of total protein [20] and sandwich ELISA assays for Heat shock protein (HSP) 70 and HSP60 (R&D Systems, Minneapolis, MN) [15]. Tissues from the right lower lung were snap frozen for RNA isolation [21].

### Quantitative RT-PCR

Messenger RNA was extracted from lung tissue from the right middle lobe with TRIzol (Invitrogen, Grand Island, NY). cDNA was produced from 1 mg mRNA using Verso cDNA kit (Thermoscientific, Waltham, MA). We used custom Taqman gene primers (Life technologies, Grand Island, NY) for ovine sequences for Interleukin 1β (IL-1β), IL-6, IL-8, early growth response protein 1 (Egr-1), cysteine rich 61 (Cyr61), and connective tissue growth factor (CTGF) [15], [22]. Quantitative RT-PCR was performed using iTaq Universal mix (BioRad, Hercules, CA) in a 20 µl reaction on a CFX Connect machine and software (BioRad, Hercules, CA). 18S primers (Life Technologies, Grand Island, NY) were used for the internal loading control. Results are reported as fold increase over mean for the PEEP only animals.

### Data Analysis and Statistics

Results are shown as mean (SEM). Statistics were analyzed using InStat (GraphPad, USA) using Student's *t*-test, Mann-Whitney non-parametric, or ANOVA tests as appropriate. Significance was accepted as p<0.05.

## Results

All fetuses survived the intervention and recovery period on placental support. The mean gestational age and birth weight were similar between groups (Table 1). The SI recruited a mean volume of 6.8±0.8 mL/kg with 75% of the volume achieved in the first 2 seconds. There were no differences in the tidal volumes achieved or compliances between the ventilated lambs with or without SI (Table 1). The post-mortem lung gas volumes at 40 cmH_2_O were not different between the groups.

**Table 1:** Animal characteristics, recruitment volumes, and ventilation parameters.

Group	N	BW	M∶F ratio	Recruitment volume	V_T_ 15 min	Compliance 15 min	Lung Volume at 40 cmH_2_O (Autopsy)
		kg		mL/kg	mL/kg	mL/(kg*cmH_2_O)	mL/kg
**PEEP**	4	2.9±0.3	1∶3	–	–	–	28.1±3.4
**SI**	6	3.3±0.1	4∶2	8.2±1.7	–	–	23.6±3.0
**V_T_**	8	3.2±0.1	6∶2	–	6.8±0.2	0.18±0.01	30.9±2.7
**SI+V_T_**	8	3.3±0.2	6∶2	5.9±0.6	6.8±0.3	0.18±0.01	28.0±2.5

### Cytokine and acute phase mRNA in peripheral lung

**Figure 1:**
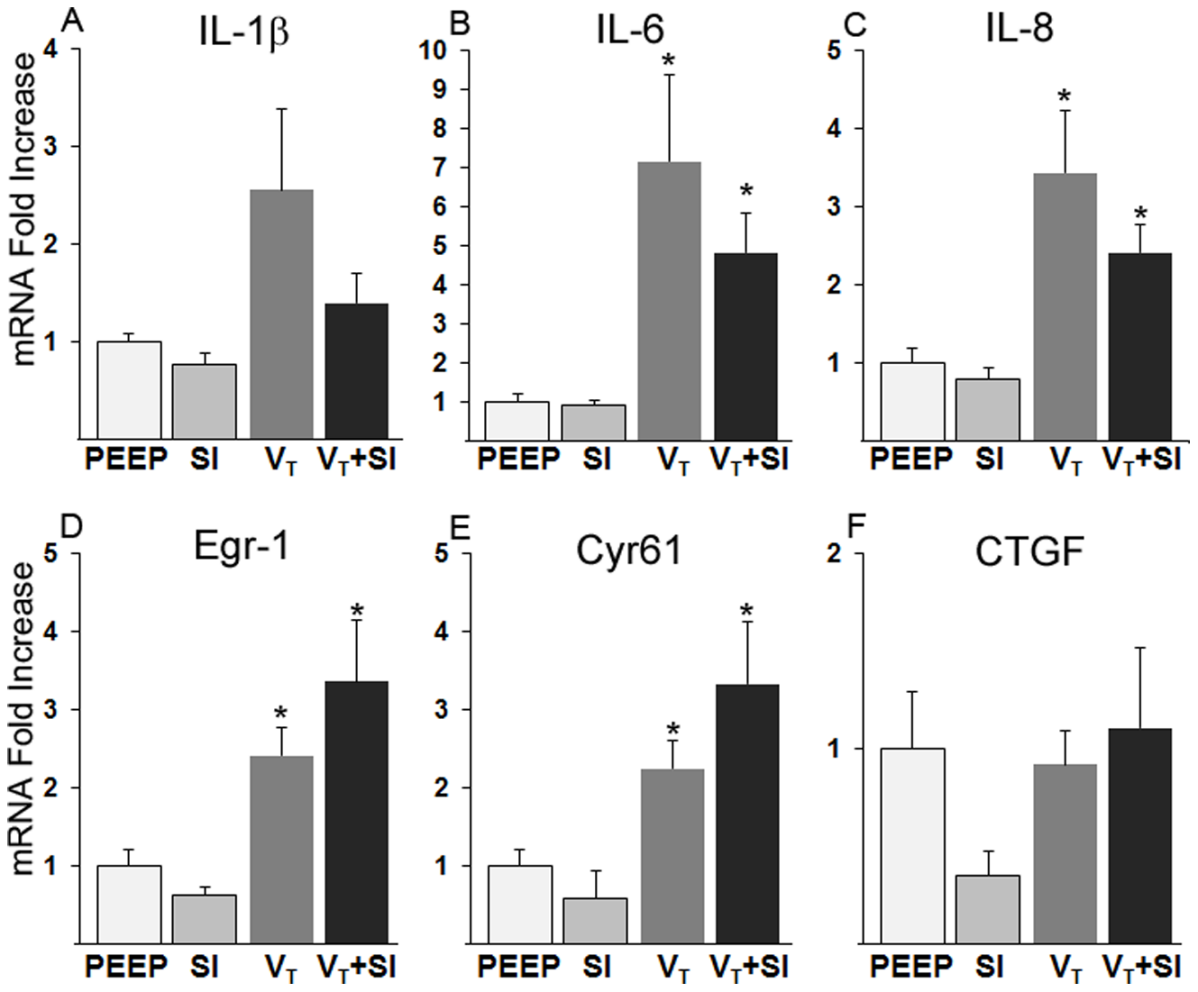
Pro-inflammatory cytokine and acute phase mRNA responses in the lung. (A) IL-1β did not increase with V_T_ (*p*>0.05). (B, C) IL-6 and IL-8 mRNA increased similarly for the V_T_ and V_T_+SI groups, with no increase with SI relative to PEEP alone. (D, E) The acute phase genes Egr-1 and Cyr61 also increased with V_T_ and V_T_+SI, with no increases with SI relative to PEEP alone. (F) CTGF mRNA did not change. *p<0.05 vs PEEP only.

Although IL-1βmRNA did not change (Figure 1A, p>0.05), IL-6 and IL-8 mRNA moderately increased in both the ventilated groups (Figure 1B, C). There were no differences between lambs receiving SI before tidal volume ventilation (V_T_+SI) and those receiving only mechanical ventilation (V_T_). Mechanical ventilation also increased the mRNA for the acute phase response genes Egr-1 and Cyr61 (Figure 1 D, E), but not CTGF (Figure 1E). There were no increases in cytokines or acute phase response mRNA with SI alone compared with animals receiving only PEEP (Figure 1).

### Fetal lung fluid and bronchioalveolar lavage fluid

FLF and BALF were evaluated for total protein, a marker of overall injury, and HSP, as indicators of acute phase proteins released into the airspace with mechanical ventilation (Table 2). Mechanical ventilation increased total protein in the fetal lung fluid at the end of period of placental support (FLF End) and in the BALF, with no differences seen in the lambs receiving V_T_ or V_T_+SI. HSP60 was increased in BALF in both V_T_ and V_T_+SI, while HSP70 was increased in the FLF end in both groups (Table 2). A SI prior to mechanical ventilation had no effect on the HSP release. SI alone did not increase total protein, HSP60, or HSP70 relative to animals receiving only PEEP.

**Table 2:** Total Protein and Heat Shock Proteins in FLF and BAL.

Group	Total Protein		HSP60		HSP70	
	FLF End ng/ml	BALF ng/kg	FLF End ng/ml	BALF ng/kg	FLF End ng/ml	BALF mg/kg
**PEEP**	232±45	37±8	1.5±0.2	35±10	46±10	2.1±0.7
**SI**	310±164	46±18	1.0±0.2	49±13	54±12	1.9±0.9
**V_T_**	1389±155*	58±14*	4.2±1.3	155±37*	135±7*	1.9±0.2
**SI+V_T_**	1618±365*	61±16*	1.7±0.6	100±15*	117±11*	1.9±0.1

Heat shock protein (HSP) Fetal Lung Fluid (FLF) Bronchoalveolar lavage fluid (BALF) Values: Mean ± SEM.

* p<0.05 vs PEEP and SI groups.

## Discussion

With the preterm fetal sheep model we tested whether a 20 second SI to 40 cmH_2_O pressure at birth would decrease lung injury from subsequent ventilation in lambs treated with surfactant. Similar to our previous experiments using higher pressures (50 cmH_2_O) in fetuses not treated with surfactant, we did not find a decrease in markers of lung injury with SI in surfactant-treated lambs [10]. However, we found that the SI alone did not cause the release of acute phase response proteins or initiate a pro-inflammatory cytokine response compared with animals receiving only PEEP [10]. We had previously shown a modest increase in the pro-inflammatory cytokines and release of HSP70 into airways with the 20 second SI at 50 cmH_2_O [10]. Concern for the negative effects of SI were previously raised when SI decreased the response to surfactant in preterm sheep [23]. The release of HSP70 and HSP60 into the airways with mechanical ventilation was not decreased with SI, suggesting that the airway stretch accompanying mechanical ventilation continued despite an attempt to open the lung with SI and achieve FRC [8], [22]. The release of HSP70 and HSP60 may have implications for lung inflammation as both are endogenous ligands for toll-like receptors [24]. The lack of an effect of SI on markers of lung injury and inflammation with or without surfactant treatment raises questions about the benefits of SI.

A 20 second sustained inflation to 40 cmH_2_O recruited a volume of only 7 mL/kg in these surfactant-treated fetal lambs. The recruitment volume was smaller than the 14.9 mL/kg we had previously achieved with a 20 sec SI at 50 cmH_2_O in fetuses not treated with surfactant, which suggests higher pressures are required to overcome the surface-tension created by the air-fluid interface, even in surfactant treated animals [9], [10]. A high pressure of 35 cmH_2_O was also needed in 36 gram newborn preterm, rabbits to achieve a FRC of 23.4 mL/kg [9]. Newborn lambs required 50 cmH_2_O to achieve 14.6 mL/kg in 20 seconds, which is similar to our previous fetal model, and lambs required 40 seconds to achieve 20 mL/kg FRC at 40 cmH_2_O [10]–[12]. Surfactant treatment only increased the volume recruited by a 20 second SI at 35 cmH_2_O from 7 ml/kg to 11 mL/kg [16]. Although the recruitment volume was similar to tidal volume used for subsequent ventilation, the use of the airtight three way slip valve allowed for the maintenance of any generated FRC prior to mechanical ventilation and created an additive effect on total open lung volume. The lung recruitment rate was more rapid with surfactant treatment than for untreated lambs. Recruitment volume achieved about 75% of the 20 sec volume by 2 seconds, whereas with the 50 cmH_2_O recruitment 50% of total lung recruitment by 2 seconds, 65% by 5 seconds, and 90% by 15 seconds [10]. These results in aggregate suggest that long SI do not achieve much more volume recruitment than a short SI.

Although our recruitment volume was lower than we expected at 7 mL/kg, it is similar to the volumes achieved in preterm infants [5], [7]. In 27 VLBW infants, Schilleman *et al.* measured an average SI volume of 8.7 mL/kg during five 3 second SIs, but most infants had a large mask leak which resulted in nearly no SI volume [5]. They also found most infants began breathing during positive-pressure ventilation and SI was most effective in breathing infants [5], [7]. In 70 VLBW infants receiving a 10 second SI at 25 cmH_2_O, von Vonderson *et al.* found that infants that did not breath recruited almost no lung volume, while those infants that breathed during the SI had an average FRC gained of 7.1 mL/kg [7].One third of the infants in this study did not recruit any SI volume, a finding the authors contributed to glottis obstruction in non-breathing infants [7]. The lambs in our study were sedated by maternal anesthesia so did not spontaneously breath, but upper airway obstruction was overcome by tracheostomy. The more uniformed aeration from a SI after surfactant in preterm sheep also supports the role of surface tension in the initial lung recruitment [16]. Since there will be great variability in surfactant pool sizes of preterm infants exposed to antenatal stressors or steroids, it is not surprising that there will be a variable FRC recruitment response to SI [5], [7], [10]. The data from our current study and other studies would indicate that SI using higher pressures may be necessary to recruit FRC in VLBW and surfactant deficient infants, a strategy that may be impractical and dangerous for surfactant sufficient infants.

Surfactant is essential for decreasing surface tension in the lung, maintaining FRC, and decreasing lung injury during mechanical ventilation of preterm rabbits and lambs [25]–[27]. At 126 days gestational age, fetal sheep are typically relatively surfactant deficient though some variability can exist. Even small changes in endogenous surfactant pool sizes can decrease lung injury from mechanical ventilation [13]. Preterm lambs with 8% of normal term surfactant pool size have decreased lung injury from mechanical ventilation, and those with greater than 5% can be maintained on CPAP alone [13], [28]. Some of the variability in recruitment volume with SI in previous studies with lambs may have been due to small changes in endogenous surfactant, although the differences could not be fully explained by surfactant pool size [10]. In an attempt to provide equal surfactant levels prior to SI, we instilled diluted surfactant via the endotracheal tube and gently mixed it into the remaining lung fluid. The positive effects of surfactant treatment were evident when we compare them with the lambs from the previous study ventilated with same tidal volume and no SI [10]. The surfactant treatment decreased the pressures needed to achieve comparable tidal volumes from 46 to 38 cmH_2_O, which increased the compliance from 0.14 without surfactant to 0.18 with surfactant (p<0.05) [10]. Surfactant treatment also decreased the mRNA expression by over 2 fold for IL-1β, Il-6, IL-8, Cyr61, and CTGF (p<0.05 for all genes vs animals ventilated without surfactant) [10]. In a similar lamb study, surfactant prior to SI decreased acute phase mRNA compared to sustained inflation followed by surfactant treatment [16]. As previously demonstrated, surfactant treatment decreases but did not eliminate the inflammatory and acute phase response to mechanical ventilation [15]. The fetal lamb model maintains placental circulation, which allows avoidance of oxidative stress with a N_2_/CO_2_ mixture, and continued ventilation, allows us to isolate the effects of SI and increases the sensitivity to detect benefits. Although surfactant treatment may have decreased injury markers, it did not unmask a beneficial effect of SI on subsequent injury.

## Conclusions

A prolonged 20 second SI at birth will recruit a FRC without altering the lung injury from continued mechanical ventilation. High pressures are needed to recruit adequate FRC, even in surfactant treated lambs. The lack of effect of SI on lung injury in both surfactant deficient and surfactant treated lambs suggests any potential benefit of a prolonged SI on intubation rates or the length of mechanical ventilation in preterm infants may not result from a decrease in the lung injury.
